# Genome Sequence of Mink Enteritis Virus Strain SD 12/01, Isolated from a Mink with Severe Diarrhea in China

**DOI:** 10.1128/genomeA.00306-13

**Published:** 2013-06-20

**Authors:** Jianke Wang, Hang Zhao, Yuening Cheng, Li Yi, Shipeng Cheng

**Affiliations:** State Key Laboratory for Molecular Biology of Special Economic Animals, Institute of Special Economic Animal and Plant Sciences, Chinese Academy of Agricultural Sciences, Changchun, Jilin, China

## Abstract

The mink enteritis virus (MEV) SD12/01 strain was isolated from a mink showing clinical and pathological signs of enteritis in Shandong, China, in 2012. The genome of MEV SD12/01 was sequenced and analyzed, which will promote a better understanding of the molecular epidemiology and genetic diversity of MEV field isolates in northern China.

## GENOME ANNOUNCEMENT

Mink enteritis virus (MEV) is a member of the genus *Parvovirus* and the family *Parvoviridae* (1). MEV is a negative-oriented, single-stranded DNA virus that causes hemorrhagic enteritis, especially among newborn and juvenile minks, with high morbidity and mortality (2). Currently, three complete genome sequences of MEVs, the mink enteritis virus strain Abashiri (Japan, 1991) and vaccine strain MEVB (China, 2009) and strain MEV/LN-10 (China, 2012), are available in GenBank.

In August of 2012, a MEV strain with high virulence, named MEV SD12/01, was isolated from a disease outbreak in a mink farm in Shandong Province, northern China. Tissue samples tested positive for MEV by PCR using primers targeting viral VP2. Sequence alignment was performed using Vector NTI 11 software (3).

The whole genome of SD12/10, excluding a partial inverted terminal repeat sequence, was amplified by PCR with four primer pairs. The PCR products were gel purified using the AxyPrep DNA gel extraction kit (Axygen) and sequenced on a 3730xl DNA analyzer (Applied Biosystems). The genomic sequence of SD12/01 comprises 4,556 nucleotides (nt) containing two open reading frames (ORFs). ORF1 (nt 100 to 2106) encodes two nonstructural proteins (NS1 and NS2), and ORF2 (nt 2200 to 4368) encodes two structural proteins (VP1 and VP2) through alternative splicing of the same mRNAs.

The genome sequence of SD12/01 shares 99.2%, 99.4%, and 99.7% nucleotide sequence identities with those of MEVB (accession no. FJ592174), Abashiri (accession no. D00765), and MEV/LN-10 (accession no. HQ694567), respectively (1, 4, 5). One deletion region (nt 4592 to 4642) was found in strain SD12/01 compared to Abashiri, which was isolated from Japan. There is an insertion region (nt 4381 to 4389) in strain SD12/01 compared to vaccine strain MEVB (China). These insertion and deletion sites are all in the 3′ untranscribed region (UTR). Even more interesting, by comparing the 3′ UTRs, we found that all MEV strains from China (including SD12/01) were missing 51 bp or 60 bp compared to Abashiri. Compared with MEV/LN-10, SD12/01 has 13 single nucleotide sequence variations: 3 variant loci were identified in the VP2 gene and resulted in 2 amino acid substitutions in the VP2 protein, and 10 variant loci were identified in the NS1 gene and resulted in 3 amino acid substitutions in the NS1 protein.

Nucleotide sequence alignments, using the Clustal V method and a BLAST search in GenBank, demonstrated that the NS1 segment of MEV SD12/01 had 98.2% to 99.8% identity among the gene sequences available in GenBank and the VP2 segment had 99.4% to 99.9% identity; all showed the highest homology with the corresponding genes from Jlin/2010 strain HQ883275, respectively. New mutations in the nonstructural protein NS1 and the structural protein VP2 have not been found yet. The strain SD12/01 is a prevalent strain in China and has the potential to be a candidate vaccine strain. The genome data of SD12/01 will be helpful for analyses of molecular epidemiology and the genetic diversity of MEV.

### Nucleotide sequence accession number.

The genome sequence of MEV strain SD12/01 has been deposited in GenBank under the accession no. KC713592.
